# A Metastable State Facilitates Low Temperature CO Oxidation over Pt Nanoparticles

**DOI:** 10.1002/anie.202423880

**Published:** 2025-01-28

**Authors:** Samantha L. Le, Christopher R. O'Connor, Taek‐Seung Kim, Christian Reece

**Affiliations:** ^1^ Rowland Institute at Harvard Harvard University Cambridge MA USA; ^2^ Department of Chemistry Tufts University Medford MA USA; ^3^ Clean Fuel Research Laboratory Climate Changing Research Division Korea Institute of Energy Research Gajeong-ro, Yuseong-gu Daejeon Republic of Korea

**Keywords:** Heterogeneous catalysis, metastable states, *in situ* DRIFTS, nanoparticles

## Abstract

The dynamic response of heterogeneous catalytic materials to their environment opens a wide variety of possible surface states which may have increased catalytic activity. In this work, we find that it is possible to generate a surface state with increased catalytic activity over metallic 2 nm Pt nanoparticles by performing a thermal treatment of the CO*‐covered Pt catalyst. This state is characterised by its ability to oxidise CO to CO_2_ at room temperature. By combining pressure pulse experiments with in situ spectroscopy we correlate the formation of this high‐activity state with the desorption of weakly bound CO* molecules from well‐coordinated Pt sites. This high‐activity state is metastable, degrading after elevated thermal treatments or upon readsorption of CO at room temperature. We conclude that this metastable state is highly localised to the surface of the nanoparticle, however its exact atomic structure remains open to speculation.

## Graphical abstract

By combining transient pressure pulse experiments with Diffuse Reflectance Infrared Fourier Transform Spectroscopy a high‐activity metastable state was identified on Pt nanoparticles, characterised by its ability to oxidise CO* to CO_2_ at room temperature. Access to this state requires a mild thermal treatment in the presence of adsorbed CO*. We hypothesise that this state is highly localised to the surface of the nanoparticle, and we find that it collapses upon elevated thermal treatments, or upon adsorption of CO at low temperature.

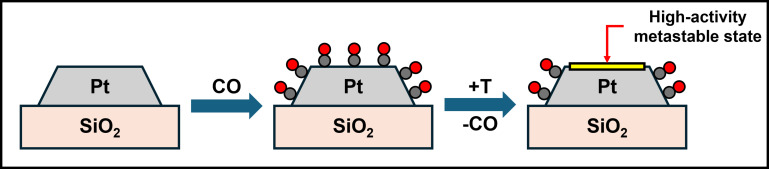

The catalytic oxidation of CO is the prototypical probe reaction in heterogeneous catalysis, and is a powerful tool for interrogating catalytic activity and structure.^[1]^ Pt catalysts have been shown to be active for low temperature CO oxidation when supported on Al_2_O_3_,[^2^, ^3^] CeO_2_,[^4^, ^5^] and TiO_2_,[^6^, ^7^, ^8^] but when supported on SiO_2_, no such activity is observed under steady‐ and non‐steady‐state conditions.^[9]^ For Al_2_O_3_‐, CeO_2_‐, and TiO_2_‐supported Pt nanoparticles, it is thought that the interaction between the Pt nanoparticles and the support generates new types of active sites (e.g., platinum carbonates or interfacial sites) that have increased CO oxidation activity. However, Pt nanoparticles supported on SiO_2_ are very weakly interacting and as such, are more analogous to freestanding Pt nanoparticles.^[10]^ The possibility of metastable sites of increased catalytic activity forming over freestanding metal nanoparticles has been proposed from theoretical calculations,[^11^, ^12^, ^13^, ^14^] but to the best of our knowledge have not been isolated experimentally. In our previous work it was observed that a pristine metallic or CO* saturated 2 nm Pt/SiO_2_ powder catalyst showed no activity for CO oxidation at room temperature. However, when CO was adsorbed at elevated temperatures, it was possible to oxidise the adsorbed CO* to CO_2_ using transient O_2_ pulses at room temperature.[^9^, ^15^] In this work, we show that this low temperature oxidation of adsorbed CO* is driven by a high‐activity state that is induced by combining CO adsorption with a mild thermal treatment at a temperature greater than 75 °C which induces partial CO* desorption regardless of whether the thermal treatment was applied under a CO environment or under vacuum. Using a combination of Temporal Analysis of Products (TAP),^[16]^ Diffuse Reflectance Infrared Fourier Transform Spectroscopy (DRIFTS), and Temperature Programmed Desorption (TPD) experiments, we are able to isolate this high‐activity state and rule out the possibilities of this phenomenon being driven by environment‐based restructuring, variations in CO* or Pt site coverage, or carbonate intermediates. Instead, we find that this metastable state is attributed to the emergence of an active Pt surface which forms after desorption of weakly bound CO* via a thermal treatment. Moreover, we find that this state is metastable and is deactivated by readsorption of CO at room temperature and by thermal treatments at elevated temperatures (>200 °C). This metastable state is highly localised to the surface of the nanoparticle, but its exact atomic structure remains unknown.

A series of Temporal Analysis of Products Temperature Programmed Oxidation (TAP‐TPO) experiments over the partially CO*‐covered 2 nm Pt/SiO_2_ catalyst find that low temperature CO oxidation is not dependent on a continuous CO gas environment (Figure 1). Pt surfaces are dynamic and are known to restructure under near ambient pressures of CO gas.[^17^, ^18^, ^19^, ^20^] Conversely, it is expected that the absence of CO gas during heating would minimise the extent of any possible surface restructuring.[^19^, ^21^, ^22^, ^23^] The influence of the CO gas environment in forming an active surface was examined by comparing a catalyst that was saturated with CO* in the presence of a continuous CO gas environment at elevated temperature (saturate) to one generated by saturating the catalyst with CO* at room temperature and subsequently heating to the same temperature under vacuum (flash). Heating alone is not expected to induce the active surface, as a pristine metallic Pt surface is generated using H_2_ pulses at 350 °C before all experiments.^[15]^ When pulsing O_2_ over the thermally treated CO*‐covered catalysts at room temperature, similar low temperature CO_2_ production rates (Figures 1a–1c) and TPO profiles (Figures 1d–1f) are observed for both the saturation (purple) and flash (red) experiments, in which CO oxidation is observed immediately at 25 °C. This demonstrates that the formation of the high‐activity state does not require a continuous CO gas environment at elevated temperature.

**Figure 1:**
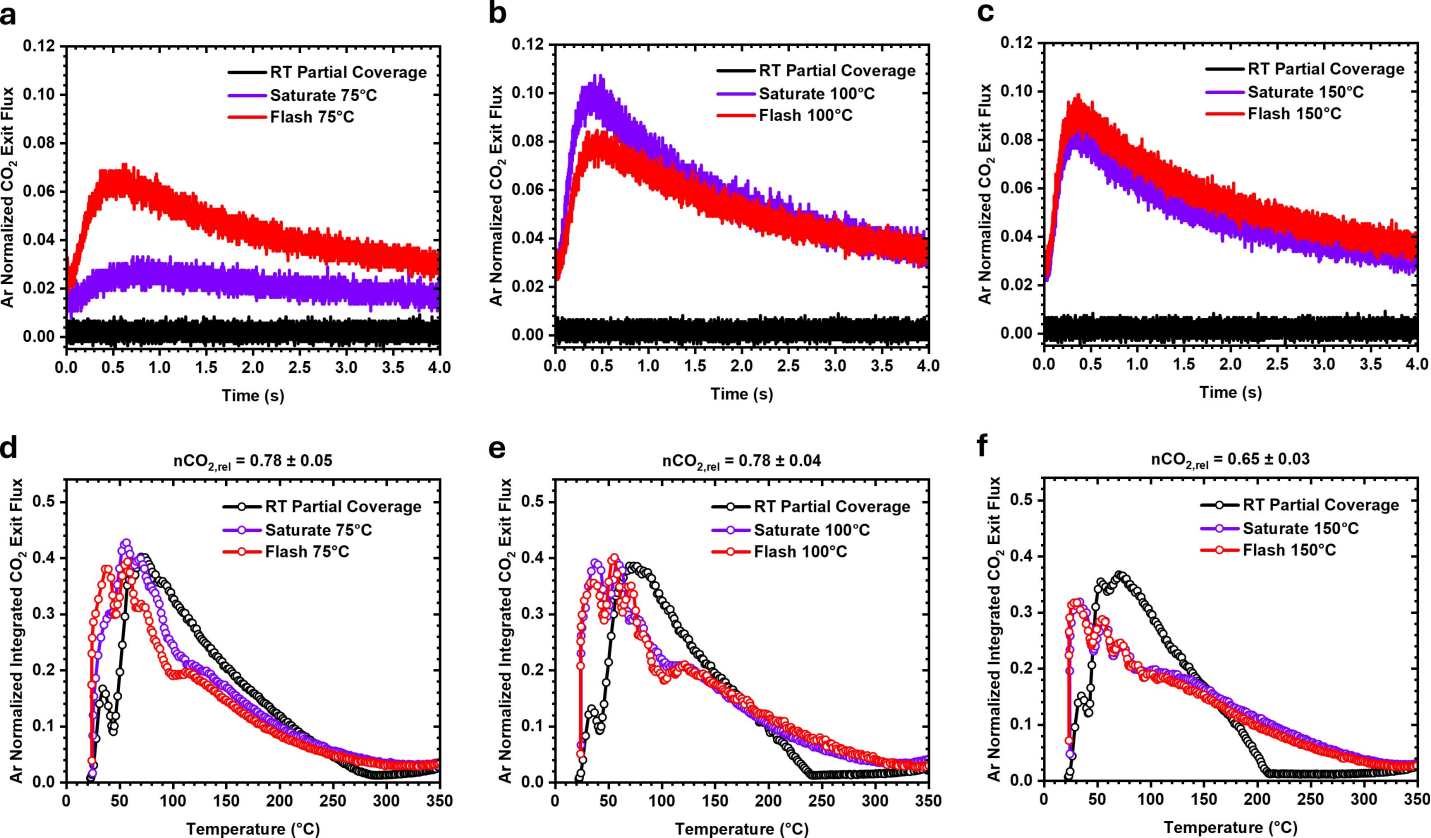
Thermal treatment in the presence of adsorbed CO facilitates room temperature CO oxidation. (**a–c**) Argon normalized m/z=44 (CO_2_) exit flux curves measured in response to an O_2_ pulse being introduced to the microreactor at 25 °C over the thermally and non‐thermally treated surfaces with similar nCO_2,rel_ values. The exit flux curves show the time dependent production of CO_2_ after the pulse of O_2_, with CO_2_ only detected over the thermally treated samples. (**d–f**) Integrated CO_2_ exit flux curves measured as a function of temperature while O_2_ is repeatedly pulsed as the catalyst is heated linearly from 25 °C to 350 °C at 8 °C/min.

The TAP‐TPO experiments were repeated over catalysts where CO was adsorbed at the same coverage as those generated from the saturation and flash experiments. As an exact CO* coverage is difficult to determine, the CO_2_ production relative to a TAP‐TPO experiment on a fully CO*‐saturated catalyst at room temperature (nCO_2,rel_) is used to estimate the initial CO* coverage. The thermally treated saturated and flashed catalysts showed similar nCO_2,rel_ values. To identify the role of the thermal treatment, a non‐thermally treated partially CO*‐covered catalyst of similar nCO_2,rel_ value was generated by performing controlled pulsing of CO at room temperature. Similar to the pristine and fully CO* saturated catalysts, the non‐thermally treated catalysts (black) showed no notable activity for CO oxidation at low temperature (Figures 1a–1c) and showed remarkably different TPO profiles (Figures 1d–1f) than those that had been thermally treated (purple and red). While baseline shifts in the CO_2_ signal (Figure S1) indicate possible CO_2_ production from 25 °C to 50 °C during the TAP‐TPO experiments over non‐thermally treated catalysts, the flat shape of the exit flux response demonstrates that this process is significantly slower than the rapid production of CO_2_ over the thermally treated catalysts. Further experiments were performed on catalysts at much lower coverages (nCO_2,rel_=0.48 and 0.29), and no instantaneous CO_2_ production was recorded at room temperature (Figure S2). The significantly reduced activity seen over non‐thermally treated catalysts of varying CO* coverage demonstrates that the increased catalytic activity over thermally treated catalysts is not due to variations in CO* or Pt site coverage.

To further rationalise the nature of adsorbed CO* sites, DRIFTS measurements were conducted to better understand the possibility of the high‐activity state being site specific. For CO* binding onto Pt, absorbances between 2100 cm^−1^ and 1872 cm^−1^ were attributed to linearly bound sites while peaks between 1850 cm^−1^ and 1621 cm^−1^ were assigned as multi‐bound (MB) sites.[^9^, ^24^, ^25^] Among the linearly bound sites, CO* bound to well‐coordinated (WC) sites appeared at wavenumbers between 2071 cm^−1^ and 2053 cm^−1^, while CO* bound to under‐coordinated sites (UC) appeared as a shoulder off the WC peaks between 2038 cm^−1^ and 1897 cm^−1^_._[^9^, ^15^, ^24^, ^26^, ^27^, ^28^] When saturating the catalyst with CO* at room temperature using pulses of CO, DRIFT spectra demonstrate that all sites are populated uniformly (Figure 2a) during adsorption. Within the first 6 pulses, the coverage of all sites was shown to increase linearly with no specific preference (Figure 2b) followed by a gradual increase in the absorbance related to the linear sites. These measurements show that during adsorption over a non‐thermally treated catalyst, there is no site specificity, with sites being filled randomly. This demonstrates that the increased catalytic activity over thermally treated surfaces is not due to opening up of vacancies of a specific CO* adsorption site.

**Figure 2:**
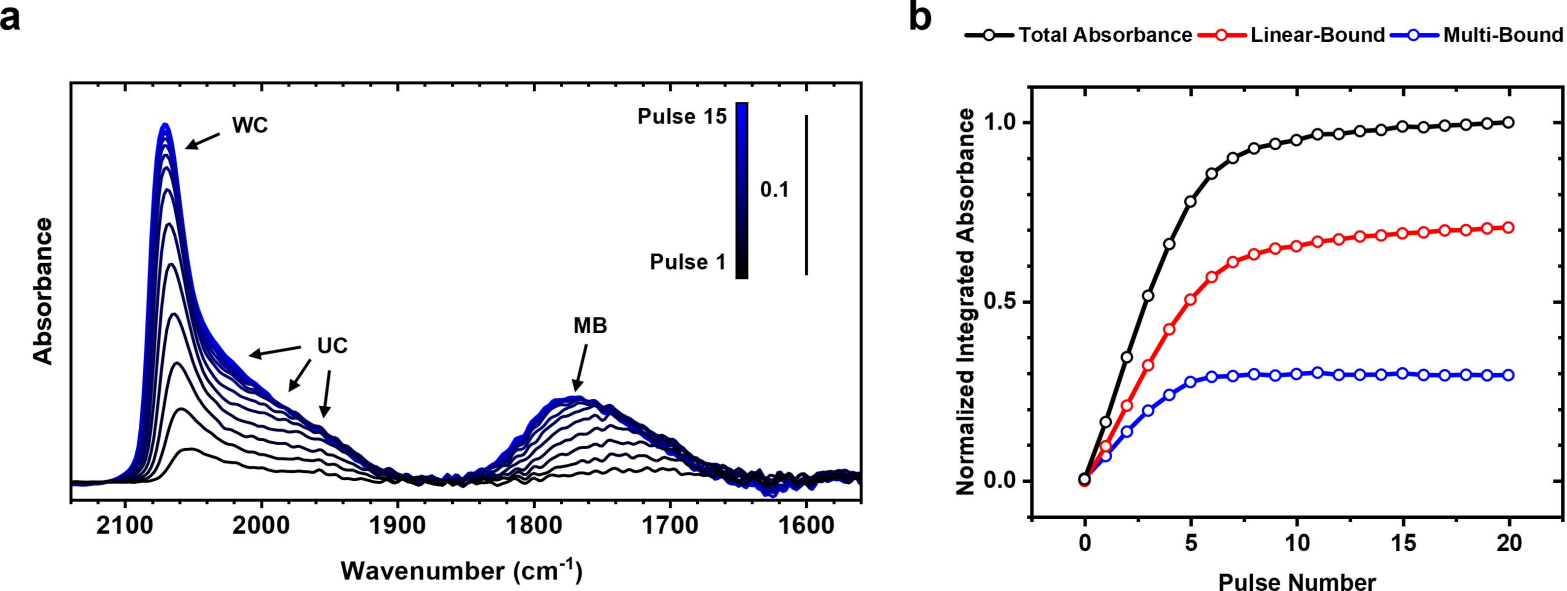
Lack of site‐specificity for CO adsorption on non‐thermally treated Pt/SiO_2_. (**a**) Consecutive DRIFT spectra between CO gas pulses under ambient pressure flow at 25 °C over a pristine, non‐thermally treated 2 nm Pt/SiO_2_ catalyst. (**b**) Integrated absorbance of the DRIFT spectra in (**a**) and deconvoluted absorbance of linear bound over well‐coordinated (WC) and under‐coordinated (UC) sites and multi‐bound CO (MB) as a function of CO pulse number. The integrated absorbances are normalized by the area of the CO*‐saturated spectra.

A series of Temperature Programmed Desorption (TPD) experiments using the TAP and DRIFTS find that the formation of the high‐activity state is correlated with the removal of weakly bound CO*, rather than partial population of a specific adsorption site. The TAP‐TPD experiments of the CO*‐covered, non‐thermally treated catalysts exhibited an initial low temperature peak centred at 35 °C (β_1_) followed by a broader, higher temperature peak centred at 276 °C (β_2_) (Figure 3a, black). The TPD experiment was limited to 375 °C to reduce the risk of sintering the catalyst, but complete desorption of CO is expected at these temperatures as confirmed by DRIFTS (Figure S3). The peak β_2_ is consistent with TPDs conducted on polycrystalline Pt, which was shown to appear regardless of CO* coverage.^[29]^ A lower temperature peak was observed on polycrystalline Pt at ~150 °C, which was attributed to lateral interactions between CO* molecules at coverages above 0.5; however, β_1_ was observed at a much lower temperature and seemed to be independent of coverage so long as the catalyst was not thermally treated. Moreover, while TPD profiles of fully CO*‐saturated surfaces of Pt/Al_2_O_3_ ^[30]^ and Pt/CeO_2_ ^[31]^ at room temperature showed the desorption of CO* between 127 °C and 280 °C, lower temperature desorption consistent with β_1_ was not observed. For a catalyst with a partial coverage of CO* that does not exhibit the high‐activity state (Figure 3a, grey), β_1_ is present, but at a significantly lower in intensity which indicates partial population of the β_1_ weakly bound CO* sites. For a catalyst that was saturated with CO* at room temperature and flashed to 75 °C that exhibits the high‐activity state (Figure 3a, green), β_1_ was not present in the TPD profile, leaving only β_2_. The β_2_ peak from the thermally treated catalyst was similar to those from the non‐thermally treated catalyst, indicating that while thermal treatment removed the loosely bound CO* attributed to β_1_, it was unable to remove those that were more stably bound seen in β_2_. Therefore, we conclude that partial population of β_1_ sites does not induce a high‐activity state, but instead it appears that the β_1_ sites must be first populated, then removed via a thermal treatment, in order to generate the high‐activity state.

**Figure 3:**
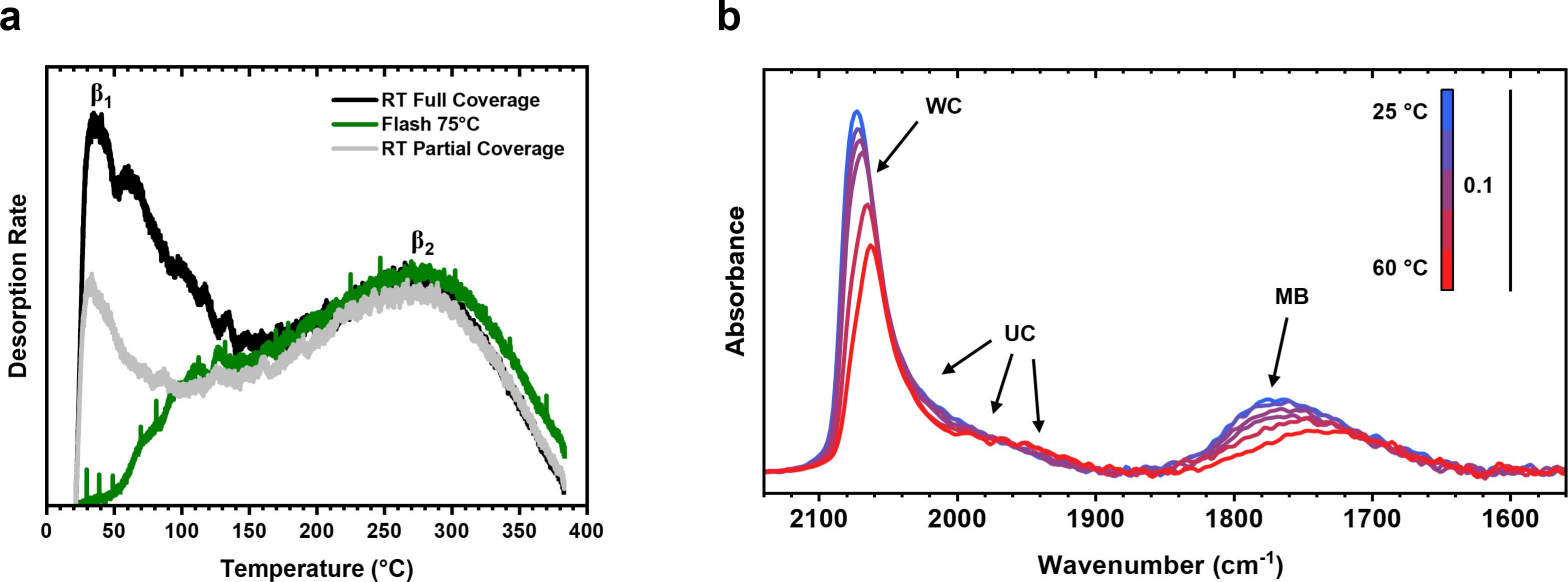
Site‐specific CO desorption of thermally treated catalysts. (**a**) TAP‐TPD experiments showing CO desorption from the non‐thermally treated catalyst (full coverage, partial coverage) and the thermally treated catalyst. (**b**) DRIFTS‐TPD experiment in which the catalyst was saturated with CO at 25 °C before being heated to 350 °C, only the DRFIT spectra until 60 °C are shown for clarity.

A DRIFTS‐TPD experiment over a catalyst saturated with CO at room temperature showed that when heating from room temperature to 60 °C, predominantly linearly bound well‐coordinated CO sites and multi‐bound CO sites were lost, while linearly bound under‐coordinated sites remained (Figure 3b). Previous work has shown that over well‐coordinated Pt(111) and Pt(100) surfaces, linear and multi‐bound CO have similar binding strengths,^[32]^ with the Pt coordination having a much larger effect on CO binding strength than bonding configuration. As we see desorption of linear‐bound CO from well‐coordinated sites, but not under‐coordinates sites at low temperatures, we attribute the low temperature peak in the TPD (Figure 3a, β_1_) to the removal of CO bound weakly to well‐coordinated sites and the higher temperature peak (Figure 3a, β_2_) to CO bound more strongly to under‐coordinated and well‐coordinated sites. Further, the absence of features at ~1600 cm^−1^ indicates no formation of Pt carbonates.[^3^, ^4^] Unfortunately, the poor thermal control in the DRIFTS cell meant that it was not possible to study the thermally treated catalysts at room temperature due to continuous desorption during the long time required to cool the sample back to room temperature. However, the DRIFTS‐TPD suggests that the low temperature peak in the TPD experiments corresponds to the removal of weakly bound linear and multi‐bound CO from well‐coordinated sites. Specifically, it is the removal of the CO* bound to these well‐coordinated sites that forms the high‐activity state.

To determine the stability of the high‐activity state in CO environments, TAP‐TPO experiments were performed over a thermally treated catalyst that was resaturated with CO at room temperature. After resaturating the thermally treated catalyst with CO at room temperature, no enhanced low temperature CO oxidation was observed (Figure 4a), and the TPO profiles were exactly the same as that of a catalyst saturated with CO at room temperature (Figure 4b). Further, TPD experiments over a thermally treated catalyst with adsorbed ^12^CO that had been resaturated with ^13^CO showed similar TPD profiles for ^12^CO and ^13^CO (Figure 4c), indicating that CO readsorption after thermal treatment generates both β_1_ and β_2_ sites (Figure 3a), and the high‐activity state is removed. The thermal stability of the high‐activity state was assessed by saturating the catalyst with CO at 350 °C. No instantaneous CO_2_ production is observed at room temperature (Figure S4), meaning that the high‐activity state is no longer present. This would indicate that the high‐activity state is thermodynamically unfavoured, most likely restructuring back to the pristine metallic state at elevated temperatures.

**Figure 4:**
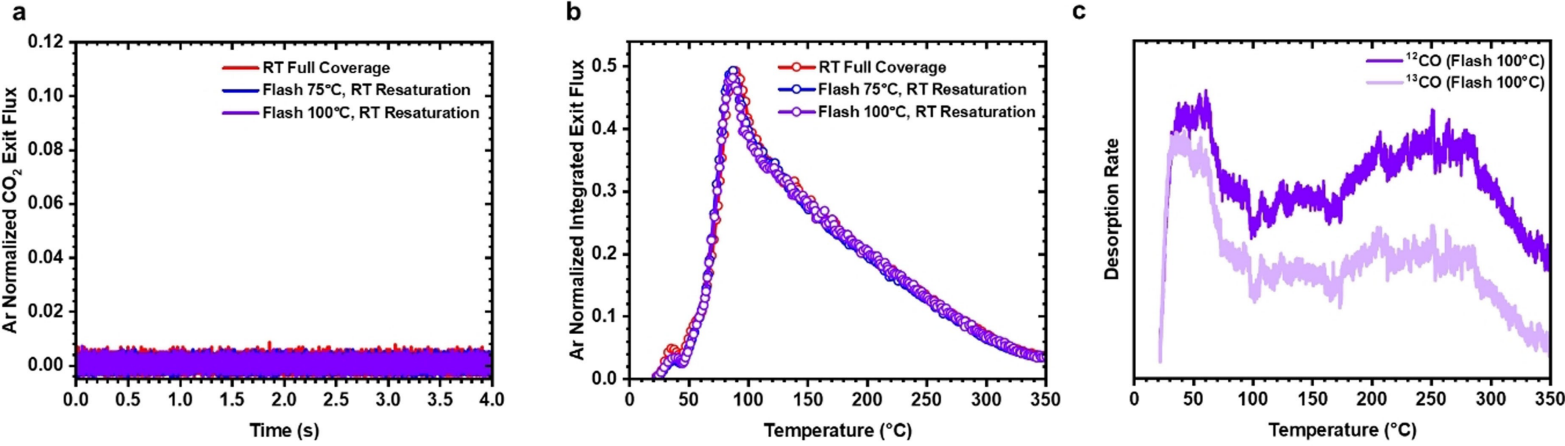
The high‐activity state is removed by readsorption of CO at room temperature. Following the thermal treatment, the catalyst was resaturated with CO at room temperature. (**a**) Argon normalized m/z=44 (CO_2_) exit flux curves after pulsing O_2_ at 25 °C over the saturated and resaturated Pt catalyst. (**b**) Full TAP‐TPO profiles for all experiments from 25 °C to 350 °C. (**c**) Isotopic‐labelling TPD experiment after the catalyst was saturated with ^12^CO at room temperature, flashed to 100 °C, and resaturated with ^13^CO at room temperature showing complete isotopic scrambling.

We had previously hypothesized that the enhanced low temperature CO oxidation observed after CO treatments at elevated temperatures over the 2 nm Pt/SiO_2_ catalyst was due to the opening of active sites by the desorption of CO*. These newly vacant sites would then provide space for O_2_ to dissociate and immediately react with CO* independent of temperature. Herein, we demonstrate that the presence of vacancies on the catalyst surface at room temperature does not result in enhanced low temperature CO oxidation and is instead induced by the formation of a metastable state after adsorption of CO and a mild thermal treatment. Non‐thermally treated catalysts of varying coverages do not exhibit enhanced room temperature oxidation activity (Figures 1, S1, S2), even with partial population of weakly‐bound CO sites (Figure 2). Rather, the high‐activity state is formed via desorption of the weakly bound CO from the well‐coordinated sites (Figure 3). Further, we show that this high‐activity state is metastable and can be removed by readsorption of CO (Figure 4) at room temperature, or by thermal treatment at elevated temperatures (Figure S4). This process is outlined schematically in Figure 5.

**Figure 5:**
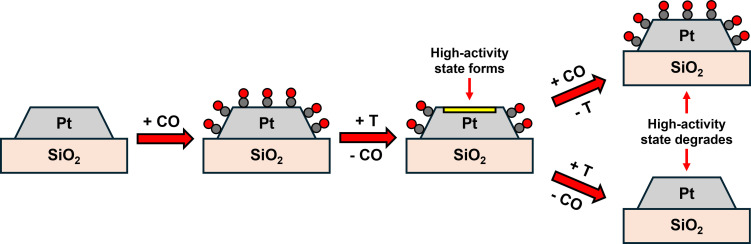
Schematic demonstrating how the high‐activity state is formed via a combination of CO adsorption and desorption over well‐coordinated sites and is degraded by further reabsorption by CO at room temperature and extended thermal treatments.

The exact atomic structure of this metastable surface state remains uncertain. Our previous studies have demonstrated that this 2 nm Pt/SiO_2_ catalyst does not sinter under the conditions employed in this work.[^9^, ^15^] Moreover, a combined DRIFTS‐XAS‐MD study^[33]^ of this 2 nm Pt/SiO_2_ catalyst heated in a CO environment found a consistent Pt−Pt bond length and coordination number across different temperatures, as evidenced by XAS. Conversely, the DRIFTS showed significant shifts in the CO vibrational frequencies with increasing temperatures, indicating a change in structure. MD simulations resolved these disparate measurements, revealing a layer of disordered surface atoms atop a bulk‐like FCC lattice structure. These findings suggest that the enhanced catalytic activity is not attributable to disintegration or alterations in the bulk structural composition of the nanoparticles. Instead, the increased activity likely stems from surface‐specific phenomena. However, the MD model was not able to study reactivity, so at present it is not possible to directly relate the metastable site to a specific surface arrangement.

We suggest that future research focus on unravelling the complex nature and formation mechanisms for this metastable state. While the role of CO in forming the metastable state is well‐established, the role of temperature is still uncertain. Both an adsorption and desorption event are required to form the metastable state, with desorption induced by a mild thermal treatment. Experiments using photoexcitation at a wavelength of 440 nm can be used to induce desorption of CO^[28]^ which could help resolve the temperature dependence, but care should be taken to not induce thermal heating from the light source. Although in situ TEM could potentially resolve the bulk atomic structure of the nanoparticles,^[34]^ the atomic structure of this highly surface‐specific and unstable state would be very difficult to resolve using currently existing in situ TEM and STEM methods due to limitations such as beam damage, or time resolution.^[35]^ The continued development of techniques to identify and characterise metastable states is crucial to the design of catalyst materials with high‐activity structures and desirable properties, as well as for understanding the activity of catalysts already in use. These proposed studies could provide valuable insights into the complex surface dynamics governing catalytic processes and pave the way for more efficient catalyst design.

Experimental procedures and supplementary Figures consisting of exit flux curves from TAP‐TPO experiments are included in the Supporting Information.

## Notes

The authors declare no competing financial interest.

## Author Contributions

The manuscript was written with contributions from all authors.

## Conflict of Interests

The authors declare no conflict of interest.

## Supporting information

As a service to our authors and readers, this journal provides supporting information supplied by the authors. Such materials are peer reviewed and may be re‐organized for online delivery, but are not copy‐edited or typeset. Technical support issues arising from supporting information (other than missing files) should be addressed to the authors.

Supporting Information

## Data Availability

The data that support the findings of this study are available from the corresponding author upon reasonable request.
