# Supplementary material for: A Metastable State Facilitates Low Temperature CO Oxidation over Pt Nanoparticles
Source: Angew Chem Int Ed Engl. 2025 Jan 28;64(14):e202423880. doi: 10.1002/anie.202423880 (PMC11966681; doi:10.1002/anie.202423880)
Supplement: Supplementary file 1 — Supporting Information [file ANIE-64-e202423880-s001.pdf]

Angewandte Chemie
Eine Zeitschrift der Gesellschaft Deutscher Chemiker

Supporting Information

A Metastable State Facilitates Low Temperature CO Oxidation over Pt Nanoparticles

*S. L. Le, C. R. O'Connor, T.-S. Kim, C. Reece**

A Metastable State Facilitates Low Temperature CO Oxidation over Pt Nanoparticles

Samantha L. Le,[a,b] Christopher R. O'Connor,[a] Taek-Seung Kim,[a,c] and Christian Reece[a*]

a) Rowland Institute at Harvard, Harvard University, Cambridge, MA, USA
b) Department of Chemistry, Tufts University, Medford, MA USA
c) Clean Fuel Research Laboratory, Climate Changing Research Division, Korea Institute of Energy Research, Gajeong-ro, Yuseong-gu, Daejeon, Republic of Korea

*Corresponding Author: christianreece@fas.harvard.edu

Experimental Methods

Catalyst Preparation. The synthesis and preparation of the 2nm Pt/SiO_2 catalyst used in this work has been described in detail previously,[1] but is briefly summarised here. The 2nm Pt nanoparticles were synthesised using a conventional colloidal method,[2] which were then deposited on a nonporous SiO_2 powder. The resulting 2nm Pt/SiO_2 catalyst was calcined in air in a tube furnace, ramping from room temperature to 500 °C in air at a ramp rate of 2 °C/min, then holding at 500 °C for one hour to remove the majority of the carbonaceous capping agent. The calcined catalyst was then reduced by flowing 10% H_2 balanced in Ar while heating from room temperature to 300 °C at 2 °C/min, generating the as prepared catalyst. Before all experiments, a consistent clean metallic (prinstie) catalyst surface is generated using a combination of pulsed O_2 and H_2 treatments over the catalyst at 350 °C in the TAP reactor which is sufficient to remove all carbonaceous species on the surface.[3] This catalyst has been shown to be highly reproducible, with the pristine metallic catalyst surface being able to be reliably reproduced, and with no sintering expected for all conditions used in this work.[1]

Temporal Analysis of Products experiments. The Temporal Analysis of Products (TAP) experiments were performed using a home-built TAP-type reactor that has been described in the previously literature.[4] A quartz tube microreactor (4 mm diameter, 64.1 mm height) was packed in the "thin-zone" configuration,[5] first with commercial sand (556.89 mg, 29.7 mm; 50-70 mesh SiO_2; Sigma-Aldrich) followed by the SiO_2-supported 2nm Pt catalyst (5.26 mg) and another layer of sand (626.51 mg, 29.7 mm; 50-70 mesh SiO_2). During a TAP experiment, a nanomole pulse of gas is sent at the entrance of the quartz microreactor which then diffuses through the bed via Knudsen diffusion. The shape of the exit flux curve, relative to the Ar inert gas, provides precise insight into the kinetics of the catalytic reaction.[6] Gas species exiting the quartz tube are detected by a mass spectrometer. To quantify the number of molecules, the exit flux is integrated to provide a

M_0 for each pulse. For all gases aside from Ar, the M_0 values were normalized to the M_0 of the Ar tracer to yield the Ar normalized integrated exit flux. $nCO_{2,rel}$ was calculated by normalizing the sum of the Ar normalized integrated exit fluxes of each experiment to the sum of the Ar normalized integrated exit fluxes of a non-thermally treated, full CO* coverage TAP-TPO experiment. Ar normalized exit fluxes were achieved by taking individual pulses as a function of time and normalizing the pulse to the integrated exit flux of Ar within the same pulse set. All gas mixtures used are balanced in Ar.

Temporal Analysis of Products Temperature-programmed oxidation experiments (TAP-TPO). All TAP-TPO experiments were performed in the home-built TAP reactor described previously. Prior to each experiment, the catalyst was reduced by pulsing a mixture of 10% H_2 (168 μs pulse width) at 350 °C for 30 minutes to achieve a pristine, metallic surface.[3] For the non-thermally treated experiments, CO was pulsed at room temperature, varying the number of pulses for different initial CO* surface coverages, before titrating with O_2 while ramping from room temperature to 350 °C at 8 °C /min. Two types of thermal treatment were applied. For the saturation experiments, CO was pulsed over the catalyst at an elevated temperature until saturation before stopping CO pulsing and cooling down to room temperature. For thermally treated flash experiments, CO was pulsed over the catalyst at room temperature until saturation and then heated to an elevated temperature under UHV with no CO pulsing. Once the target temperature was reached, the heating was turned off and the catalyst was left to cool back to room temperature. Following both types of thermal treatments, the TPO experiment was performed where a mixture of 20% O_2 was repeatedly pulsed over the catalyst while heating from room temperature to 350 °C at 8 °C /min.

Temporal Analysis of Products temperature-programmed desorption (TAP-TPD) experiments. Prior to each experiment, the catalyst was reduced with using pulses of 10% H_2 at 350 °C for 30 minutes to ensure a metallic surface. For the TAP-TPD experiments, all CO surface conditions were applied in the same method as described in the TAP-TPO experiments. The temperature was then ramped from room temperature to 350 °C at 15 °C /min with no pulsing under UHV. The desorption of CO was measured using a mass spectrometer ($m/z = 28$). For TPD experiments involving ^{13}CO, the catalyst was first saturated with CO at room temperature before being flashed to an elevated temperature. Then, the sample was brought back down to room temperature and resaturated with ^{13}CO. With no pulsing, the temperature was increased from room temperature to 350 °C at 15 °C /min while tracking the desorption of both CO ($m/z = 28$) and ^{13}CO ($m/z = 29$).

Diffused Reflectance Infrared Fourier Transform Spectroscopy experiments. DRIFTS experiments were performed in a low-volume reaction chamber (Harrick

Scientific), equipped with ZnSe windows using a Praying Mantis accessory (Harrick Scientific) to mount the reactor in a Bruker Invenio FT-IR spectrometer with a liquid nitrogen cooled HgCdTe (MCT) detector. The FT-IR spectrometer and Praying Mantis accessory were constantly purged with dry N_2 produced from compressed air by a purge gas generator (Parker, Spectra 15). The DRIFTS reactor was packed by sequentially loading a 304 stainless-steel mesh (150 × 150 mesh), approximately 140 mg SiC, and approximately 8 mg of sieved Pt/SiO_2. The thermal gradient between the catalyst surface temperature and the measured thermocouple was calibrated by an optical pyrometer using an emissivity of 0.95.[3,7] All DRIFTS measurements were collected using 200 scans with a resolution of 4 cm^{-1} under a total volumetric flow rate of 25 sccm controlled using a home-built rapid gas switching system.[8] To probe the possible site-specific CO adsorption on 2nm Pt/SiO_2 during room temperature saturation, quick switches were performed at 25 °C to mimic the pulsing in the TAP experiments. The series of switches were between one line consisting of Ar (25.00 sccm) for 0.5 s and a second gas line consisting of 20% CO in Ar (0.25 sccm) and 1% Kr in Ar (24.75 sccm) for 60s. To observe the population of active sites during room temperature adsorption, sequential DRIFT spectra were recorded in the pure Ar gas environment between the switches. A DRIFTS-TPD experiment was also performed in the DRIFTS reactor in which the 2nm Pt/SiO_2 catalyst was saturated at room temperature using the CO gas mixture above. To monitor the site-specific desorption of CO, consecutive DRIFT spectra were taken in pure Ar as the sample was heated from 25 °C to 350 °C using a heating rate of 16 °C/min.

Supplementary Figures

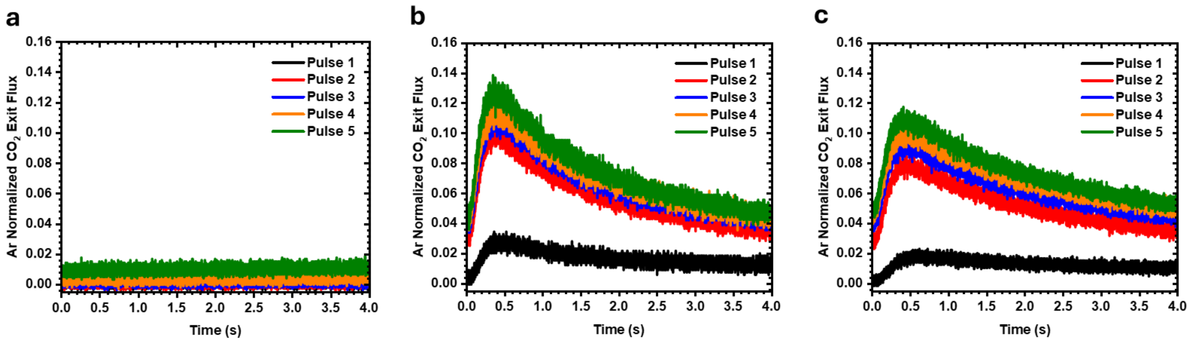

Fig. S1. Initial CO_2 production at 25 °C for the TAP-TPO experiments with 100 °C thermal treatments show enhanced low temperature CO oxidation only after thermal treatment. (**a-c**) Argon normalized CO_2 exit fluxes from TAP-TPO experiments in which the surface was either (**a**) partially covered at room temperature, (**b**) saturated with CO at 100 °C, or (**c**) saturated with CO at room temperature and flashed to 100 °C. $nCO_{2,rel}$ values for the thermally treated experiments are similar, and these are the same experiments shown in **Fig. 1b, e.**

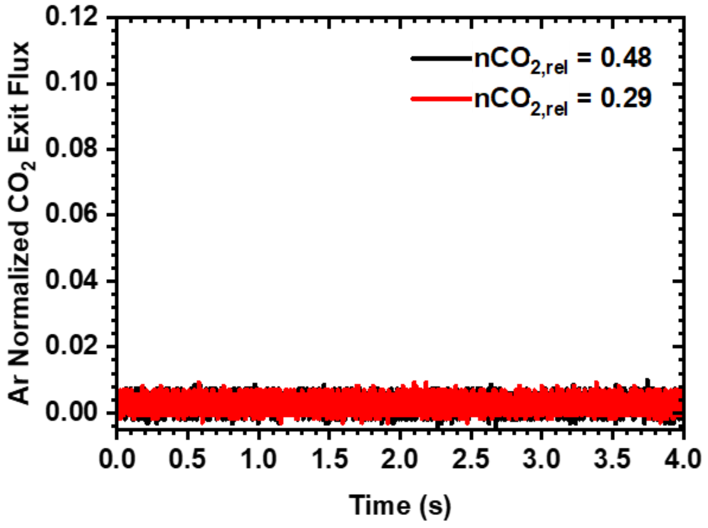

Fig. S2. No enhancement of low temperature CO_2 production occurs at low CO* coverages without thermal treatment. Argon normalized CO_2 exit fluxes at 25 °C for non-thermally treated TPOs of the lowest performed CO coverages.

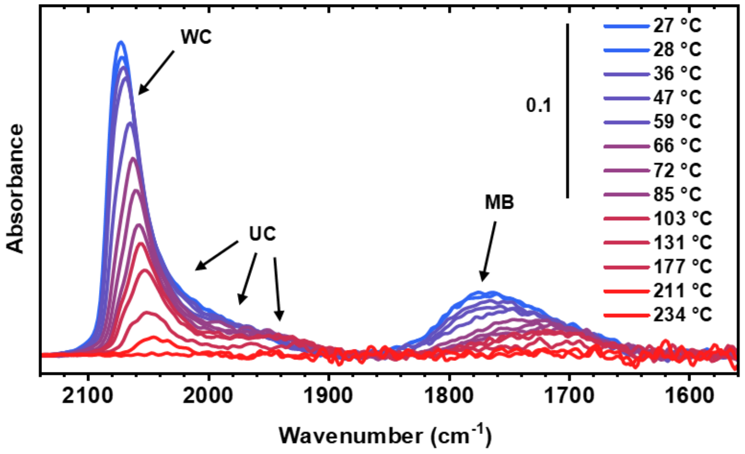

Figure S3. DRIFTS-TPD experiment in which the catalyst was saturated with CO at 25 °C before being heated to 350 °C. Complete CO desorption occurs by 234 °C in the DRIFTS cell.

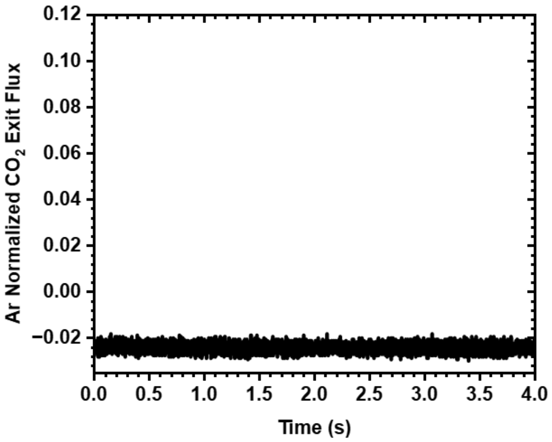

Fig. S4. CO_2 production at 25 °C after a surface was saturated with CO at 350 °C shows no enhancement of room temperature CO oxidation. Argon normalized exit flux for m/z = 44 (CO_2) at 25 °C during a TAP-TPO experiment after the surface was saturated with CO at 350 °C.

References

(1) Kim, T.-S.; O'Connor, C. R.; Le, S. L.; Reece, C. A Well-Defined Supported Pt Nanoparticle Catalyst for Heterogeneous Catalytic Surface Science. *J. Mater. Chem. A* **2024**.

(2) Song, H. C.; Oh, S.; Kim, S. H.; Lee, S. W.; Moon, S. Y.; Choi, H.; Kim, S.-H.; Kim, Y.; Oh, J.; Park, J. Y. The Effect of the Oxidation States of Supported Oxides on Catalytic Activity: CO Oxidation Studies on Pt/Cobalt Oxide. *Chem. Commun.* **2019**, *55* (64), 9503–9506.

(3) Kim, T.-S.; O'Connor, C. R.; Reece, C. Interrogating Site Dependent Kinetics over SiO2-Supported Pt Nanoparticles. *Nat Commun* **2024**, *15* (1), 2074.

(4) Brandão, L.; High, E. A.; Kim, T.-S.; Reece, C. Simplifying the Temporal Analysis of Products Reactor. *Chemical Engineering Journal* **2023**, *478*, 147489.

(5) Shekhtman, S. O.; Yablonsky, G. S.; Chen, S.; Gleaves, J. T. Thin-Zone TAP-Reactor - Theory and Application. *Chemical Engineering Science* **1999**, *54* (20), 4371–4378.

(6) Gleaves, J. T.; Yablonskii, G. S.; Phanawadee, P.; Schuurman, Y. TAP-2: An Interrogative Kinetics Approach. *Applied Catalysis A: General* **1997**, *160* (1), 55–88.

(7) Meunier, F. C. Pitfalls and Benefits of in Situ and Operando Diffuse Reflectance FT-IR Spectroscopy (DRIFTS) Applied to Catalytic Reactions. *React. Chem. Eng.* **2016**, *1* (2), 134–141.

(8) High, E. A.; Lee, E.; Reece, C. A Transient Flow Reactor for Rapid Gas Switching at Atmospheric Pressure. *Rev. Sci. Instrum.* **2023**, *94* (5), 054101.
